# An action-observation method for studying social perception: a mini-review

**DOI:** 10.3389/fpsyg.2024.1473498

**Published:** 2024-09-25

**Authors:** Manlu Liu, James T. Enns

**Affiliations:** Department of Psychology, University of British Columbia, Vancouver, BC, Canada

**Keywords:** social perception, action perception, selective attention, joint action, social cognition

## Abstract

An important aspect of any social interaction involves inferring other people’s mental states, intentions, and their likely next actions, by way of facial expression, body posture, eye gaze, and limb movements. An actor’s *production* of actions during social interactions and the observer’s *perception* of these actions are thus closely linked. In this review, we outline an action-observation methodology, which not only allows for separate analyses of production and perception, but also promotes the study of the dynamic interaction between these two sides of every social exchange. We review two lines of research that have benefited from its application. The first line focuses on individuals performing tasks alone and the observation of their actions by other individuals in order to make inferences about their attentional states. The second line of study focused on pairs of individuals performing collaborative tasks in naturalistic settings and the observation of these performances by other individuals. We offer several suggestions for how this methodology can be extended to improve on the limitations of the present studies, as well as some suggestions of how to use this methodology to venture into new territory. Our aim is to inspire future research applications of this methodology in order to advance our understanding of social action production and perception.

## 1 Introduction

Social interactions almost always involve non-verbal communication in addition to the verbal exchanges we tend to focus on in our conscious experience. Bodily signals from facial expressions, limb gestures, body posture, and eye gaze are all produced naturally in most interactions and can be used to infer others’ mental states, intentions, and the likely actions they may take next (Siegman and Feldstein, 2014). The human ability to produce these non-verbal signals, on the one hand, and then to perceive them on the other, is essential for successful social interactions. In this review, we will outline a methodological process for studying both the actors’ *production* of actions during social interactions and the *perception* of these actions by observers. We refer to this as an *action-observation* methodology.

Our interest in studying both sides of a social exchange — i.e., the signals generated by an actor and the messages received by an observer — are fueled by two emerging trends in the study of social perception. One is the call for research to be more ecologically valid (Kingstone et al., 2008). There is now a rapidly growing trend to study perception and cognition ‘in the wild,’ rather than simply hoping that tasks studied in the laboratory will somehow generalize to the everyday behaviors that researchers are trying to understand. Of course, a study of behavior under more naturalistic conditions means that it will necessarily lack many of the controls one expects to see in a laboratory study. But for proponents of this view, these limitations of control are readily offset by the benefits that come from measuring the variability inherent in everyday settings. They see variability as the important data that needs to be understood, rather than as a nuisance that needs to be suppressed. The cost of not being able to make strong causal statements is more than made up for by the benefit of measuring behavior that occurs naturally.

A second emerging trend that provides context for our review is a desire to study the ‘interactive reciprocity’ inherent in human behavior, something that is missing when we study actors or observers in isolation from one another (e.g., see Ristic and Capozzi’s, 2022, special issue of *Visual Cognition*). In the realm of human eye movements, Risko et al. (2016) have referred to the ‘dual function of gaze’ in natural social situations, where the eyes have both an encoding (information acquisition) and a signaling function (social communication of one’s mental states). Traditional methods fail to capture this two-way street of information processing. Schilbach et al. (2013) goes so far as advocating for a ‘second-person neuroscience,’ by which they mean the development of methods for measuring both behavior and brain function while human participants are interacting with one another. These authors call for research that directly compares social perception from an interactor’s point of view (second-person) versus from an observer’s point of view (third person).

The desire to study both side of a social exchange is consistent with the theoretical assumption that individuals form an internal model of others’ perceptual, decision, and action processes during a social interaction. This means they form internal models of the other’s attention, their mental states, and their action goals. This internal model is used in a predictive way to guide their expectations of the social other and their likely subsequent behavior (Bach and Schenke, 2017; Graziano, 2022; Graziano et al., 2020; Pesquita et al., 2018). From this theoretical assumption, it is critical that one studies the dynamic interplay between action production and action perception.

Although traditional social cognition research fails to capture this dynamic interplay, recent studies have made notable progress in that direction. Here we highlight some examples of these studies, offering our mini-review as step in the direction of measuring the dynamic interactions that occur between action production and perception in a social context. We hope it will help move us closer to answering questions such as: What is the nature of the internal models formed by collaborating actors? How do the models formed by observers correspond to those of the actors? Which elements of the production of social behavior are effectively modeled by observers and which are not?

At a practical level, the method involves conducting systematic research in two distinct phases. In phase one, study participants, whom we refer to as actors, engage in some activity while they are video and/or audio recorded. Social, emotional, and cognitive variables that may influence the production of the actors’ behavior can be manipulated during this phase. Actor behavior in the recordings can then be analyzed as a function of these variables, to measure their relative strength of influence. Individual differences among actors can also be assessed, in order to gain insight into the diversity of human response to the same variables.

In phase two, we enlist an independent group of participants, whom we call observers, to respond to the actor videos. By measuring observer’ responses to the actor videos, we can determine observer sensitivity to the underlying factors that shaped actor behavior. It is even possible to assess individual differences that exist in the way actors approach the task, and to do the same for observers, who also may differ from one another in their sensitivity to these actions.

In our review of the development of this methodology, we will summarize research that has benefited from its application. A summary of these studies is given in Table 1, where for each study cited, we have noted a few features to be compared for both the Phase one actor study and the Phase two observer study. Within each phase, we note the sample size, the identity of the participants, the recording method, any manipulation made to influence behavior, and whether there was some analysis of individual differences. This allows for easy comparison between studies and serves to highlight areas where future research could strengthen the existing designs.

**Table 1:** A summary of studies in the review.

Phase 1 actors				Phase 2 observers			
Task	Recording	Manipulation	Individual differences	Observational task	Medium	Social manipulation	Individual differences
Brennan et al. (2011)							
Individual young adults (Exp1 *N* = 24, Exp 2 *N* = 24)				Naive young adults (Exp1 *N* = 69, Exp 2 *N* = 59)			
Visual search	Visual	Active vs. passive strategy	Low vs. high efficiency searchers	Global ratings (energy, activity), local ratings (eye, head), mindset ratings (interest, emotion)	Video recordings	None	None
Pesquita et al. (2016)							
Individual young adults (*N* = 4)				Naive young adults (*N* = 30 in each of four experiments)			
pointing task	Visual	Externally directed vs. free choice	None	Speeded prediction of pointing direction	Video recordings	None	Autism spectrum
Chouinard et al. (2024)							
Individual young adults (*N* = 3)				Naive young adults (*N* = 70)			
Pointing task	Visual	Externally directed vs. free choice	Slow vs. fast pointers	Speeded prediction of pointing direction	Video recordings	None	Autistic vs. non-autistic
Manera et al. (2011)							
Hands of individual young adults (*N* = 8)				Naive young adults (*N* = 12)			
Reaching to grasp task	Visual	Cooperation vs. competition	None	Discriminate cooperation vs. competition	Natural video, point lights	None	None
Vinton et al. (2024)							
Full body motion capture of young adults (*N* = 4)				Naive young adults (*N* = 230)			
Everyday actions: catching a ball, petting a dog, etc.	Visual	Transitive-intransitive actions, social-nonsocial	None	Ratings of action features	Avatar powered by motion capture data	None	None
Benedek et al. (2018)							
Individual young adults (*N* = 10)				Naive young adults (*N* = 108)			
Anagram solving task	Visual	Externally vs. internally directed attention	None	Attention state discrimination	Video recordings, still photos	None	None
Liu et al. (2024)							
Individual young adults (*N* = 3)				Naive young adults (Exp 1 *N* = 147, Exp 2 *N* = 142)			
Emotion ratings of pictures	Visual	Negative vs. positive valence, low vs. high arousal	None	Facial emotion discrimination	Video recordings	Self vs. other-monitoring	Stress levels, believability
Pesquita et al. (2014)							
Pairs of professional musicians (*N* = 3)				Naïve young adults (Exp 1 *N* = 55, Exp 2 *N* = 111)			
Jazz duets	Audio	Live, studio, dubbed	None	Emotion ratings, musicality ratings, tapping	Audio recordings	None	Naïve listeners, musicians, autism spectrum
Brennan and Enns (2015a)							
Pairs of young adults (*N* = 44)				Naïve young adults (*N* = 2)			
Collaborative visual search	Audio-visual	None	Friendship ratings	Friendship ratings, utterance transcription	Audio-video recordings	None	None
Brennan and Enns (2015b)							
Pairs of young adults (*N* = 74)				Naïve young adult (*N* = 1)			
Collaborative visual search	Audio-visual	Friends vs. strangers, visible vs. partition	Stress levels (heart rate, skin conductance)	Utterance transcription	Audio-video recordings	None	None
Trevisan et al. (2021)							
Pairs of school-age children (*N* = 50)				Naïve young adults (*N* = 2)			
Table carrying	Audio-visual	Neurotypical, autism diagnosis, adult support	None	In-step synchrony ratings	Video recordings	None	None

With this guide in hand, we first of all note that the first half of the studies we review involve actors in phase one who are single individuals performing various cognitive and action tasks. The observers in phase two of these studies are participants volunteering from the same population, but who are naïve to the purposes of the study in phase one, and who are blind to the specific conditions that the actors are experiencing. These studies are thus attempting to study the people watching skills that we employ every day when making automatic and, in many cases unconscious, evaluations of others (Chen and Bargh, 1999; Ferguson and Zayas, 2009).

The second half of our review concerns studies in which the phase one actors are working in collaboration with another person. This allows us to study the production of collaborative cognition and action in settings that more closely resemble naturalistic situations and that include a ‘second-person’ perspective (Schilbach et al., 2013). By recording these sessions, we are then also able to study the perception of these social exchanges by ‘third-party’ observers.

A final section of the review looks forward, discussing extensions of the existing research that might strengthen what has already been learned. We also discuss possible applications of this methodology to two areas of social perception that have not yet been studied in this way. By explicitly laying out our assumptions and the steps in the action-observation method, along with offering examples of past work using the method, we hope to inspire future research that will improve our understanding of social perception in interactive and naturalistic settings.

## 2 Actors perform tasks individually, independent observers evaluate their performances

This section reviews four lines of research that used the action-observation method to examine the production and perception of various states of attention in others. Attention is an umbrella term used by cognitive researchers to refer to the selective nature of perceptual processes and actions. One important dimension of selective attention refers to the mental effort required to stay on task, to avoid distractions, and to efficiently coordinate perceptual processes with action outputs (Kahneman, 1973). We often assess the mental load being experienced by others in everyday life by the fluency of their actions, including their gestures and speech (Betz et al., 2023). This aspect of attention, and its perception in others, was studied in Brennan et al. (2011), by having actors perform a visual search task in phase one, while naïve observers assessed their attentional efforts in phase two.

A second important dimension of attention concerns its guidance, which is among the most widely studied topics in all of cognitive science (Corbetta and Shulman, 2002; Posner, 1980; Posner and Rothbart, 2007). Attention is said to be under endogenous control when we voluntarily decide to act on an event; it is under exogenous control when the action is governed by sudden change in the environment. This distinction is easily understood when imagining someone you are talking to reaching for their phone. Whether the person has shifted their attention to the phone voluntarily (endogenous) or whether they did so because it blinked unexpectedly (exogenous) is critical information about how their attention was controlled in that moment. This discrimination of another person’s control over their attention was studied in Pesquita et al. (2016) by having actors reach and point to one of two locations in phase one, while naïve observers attempted to guess where they would point in phase two. A related distinction between internally versus externally directed attention was studied by Benedek et al. (2018).

A third line of research examines attention to the hidden states of actors, namely the intentions behind the actions they are taking, the social standing signaled by the actions, and whether the actions are in the context of cooperation or competition (Manera et al., 2011; Vinton et al., 2024).

A fourth important dimension of selective attention concerns its close relationship with the emotional state of an observer. For example, participants in a happy mood are biased to look in the direction of positive emotional events (Wadlinger and Isaacowitz, 2006), they are more likely to process the “forest” over the “trees” (Derryberry and Tucker, 1994; Derryberry and Reed, 1998; Fredrickson, 2003; Gasper and Clore, 2002), and they perform better in time-sensitive attentional tasks (Jefferies et al., 2008). Notably, the reverse direction of influence seems just as effective, with focused attention on a task itself contributing to positive emotions. Flow theory is an account of how skilled, fluid performances in many domains of life are associated with positive emotions (Rathunde and Csikszentmihalyi, 1993). Simple environmental manipulations such as increasing stimulus clarity and contrast lead to positive emotional consequences (Reber et al., 1998). In phase one of a study, Liu et al. (2024) had actors produce facial expressions in response to emotionally evocative pictures. In phase two, naïve observers evaluated the emotional valence (positive versus negative) of the facial expressions actors made in response to these pictures. An important added feature of the Liu et al. (2024) study is that the social setting of the observers was manipulated. This allowed an examination of the link between emotion perception of the actors and the attentional state of the observers. Some observers were led to believe their character was being evaluated (biasing their attention to self-monitoring), whereas other observers believed they were in fact evaluating the actors whose expressions they were judging (biasing their attention to other-monitoring).

### 2.1 The attentional effort of visual search

Brennan et al. (2011) set out to study how much of the objective data gained from studies of humans performing visual search tasks (i.e., response times and accuracy measures gained from key presses) could be obtained by asking independent people to simply observe a searcher and to make person perception judgments. The authors’ motivation stemmed from a flurry of research in the previous decade demonstrating the remarkable phenomenon of thin-slicing (Ambady et al., 1995; Borkenau et al., 2004; Carney et al., 2007; Rule et al., 2009; Weisbuch and Ambady, 2011). Thin-slicing refers to the ability of persons to make rapid evaluations of the personality, disposition, and intent of others from very small samples of their behavior. The authors motivated their study by asking, why should cognitive researchers not also consider this potential source of information?

In phase one of Experiment 1, Brennan et al. (2011) recruited actors to complete a typical visual search task on a computer screen. On each trial, actors were presented with photos of a cluttered office and instructed to indicate the location of one of 10 common objects by indicating its screen location with a keypress. Three well-established and independent factors that influence the difficulty of these visual searches were manipulated: (1) the individual difference factor of search proficiency, which separated actors into low and high efficiency searchers based on their response speed and accuracy (2) whether the search strategy was active or passive (Smilek et al., 2006), and (3) whether the target was generally easy or hard to find in the photo. Each actors’ upper body and head were video recorded using the webcam of the computer and later edited to make video clips representing each trial in the experiment.

The results of phase one showed that actor’s search performance was significantly influenced by each of the three factors of search proficiency, search strategy, and task difficulty. A regression model that included these three variables as predictors of search speed and accuracy accounted for 68% of the variation in search performance. This set the stage to test the question of how much of the same variation in search performance could be accounted for by third-person observation of the actors performing the searches on each trial, without providing them with any information concerning the displays they were looking at or the keys they were pressing.

In phase two of the experiment, a new group of participants were recruited as observers to watch the video clips of the actors in a random order. In one condition observers were instructed to rate the visible behaviors of actors based on their global behavior using Likert scales (e.g., Proficiency: How fast and accurate is the searcher on this trial? Energy: How much physical effort is displayed on this trial? Activity: How active is the searcher on this trial?). In another condition, other observers rated the visible behaviors of actors based on the actors’ visible local behavior (e.g., How much did the actor move their head and eyes?). In a third condition, other observers rated the actors’ motivational mindset (e.g., How much interest is the actor showing? Does the actor appear pleased or satisfied?). These observers were blind to the displays the actors were viewing, the strategy condition actors had been assigned to, and whether the actor was a general low or high proficiency searcher.

The results of the ratings made in phase two showed that observers’ ratings of global behavior, local behavior, and mind-set attributions were all sensitive to actors’ search proficiency, search strategy, and task difficulty. The ratings that best predicted search performance overall were ratings of eye movement frequency and positive emotion, which accounted for 63% of the overall variance in search performance as assessed by response time and accuracy in phase one.

Experiment 2 tested the findings from Experiment 1 to a more naturalistic setting. Instead of completing the visual search task on computer, the actors completed the task in the actual office scene depicted in in the photos of Experiment 1. The aim here was to explore whether person perception might be even more accurate in a more natural setting, where actors move about with their whole bodies to find the hidden target object. The general procedure was similar to that of Experiment 1, with the exception that actors completed the task on each trial by opening the door to a real office and once inside searching and then pointing to the target object once they found it. Actors were video recorded on each trial by a camera on a tripod inside the office.

In phase two, independent observers again rated the actor on each trial using the same scaled described for Experiment 1. The results showed that ratings of person perception were sensitive to the factors of search proficiency, search strategy, and search difficulty even in this more naturalistic setting. However, there were some interesting differences in the details. For example, ratings of head movement frequency were more closely linked to these factors in the real office search (recall that ratings of eye movements were most predictive in the previous computer search). For practical reasons, there were also many fewer trials for each actor in the real office search than in the computer search, and the video resolution of the actor’s face was lower, making direct comparisons of the results difficult. Nevertheless, together with the rated activity levels on each trial, head movement ratings accounted for more than 36% of the total variance in search efficiency over the whole set of data.

Taken together, the two experiments of Brennan et al. (2011) illustrate that observers can reliably evaluate key aspects of visual search performance using only the visible behaviors of the actor, such as eye and head movements, their overall activity level, and facial expressions of emotion. This provided an early proof-of concept, that the action-perception method could be a valuable tool in furthering our understanding of the cognitive processes involved during a visual search task. The results also highlighted that important aspects of search efficiency were signaled in the visible behaviors and emotions expressed by actors during visual search.

Some of the limitations of this study are also evidence when comparing it to others in Table 1. For example, there was no manipulation of social cognition in the observers in Phase two and no analysis of the individual differences in observers’ abilities to read the signals that correlated most strongly with the actors’ performances in Phase one. These questions are ripe for further research.

### 2.2 The control of attention in action

The study by Pesquita et al. (2016) was motivated by the theoretical idea that when we view someone performing an action, the mental model we create to understand their performance does more than simply infer the spatial direction of their visual attention (Friesen and Kingstone, 1998; Langton and Bruce, 2000). They hypothesized that the model might also include information about the current state of control over attention, namely, whether attention was being directed by the intent of the actor (endogenously) or whether it was directed by external stimulation (exogenously). The hypothesis was based on a theory that social awareness involves the predictive modeling of other people’s attentional states, including the nature of its control (Graziano, 2013; Graziano and Webb, 2015).

In phase one of the study, actors sat in front of a plexiglass window holding two LED lights. On externally directed trials (exogenous control), one of the two lights illuminated randomly, and actors were instructed to reach to it as quickly as possible. On free choice trials (endogenous control), both lights were illuminated, and actors were instructed to reach to the light of their choice. Actors were video recorded during performing this task through the window. Analysis of the actors’ reaches showed that chosen reaches took longer to achieve peak acceleration and had more curved trajectories than directed reaches. The authors attributed these differences to the greater decisional uncertainty of choice reaches in comparison to directed reaches.

In phase two of the study, observers were presented with randomly selected video clips of actors from the first phase. The light of the LEDs was not visible in these clips, ensuring that the observers were blind to whether the reach was under endogenous or exogenous control. The selected clips were also equated for their overall duration, ensuring that there were no overall temporal differences between conditions. Nonetheless, when observers were instructed to predict the actors reach by pressing a key corresponding to the target’s location, they were able to respond more quickly and accurately on choice than direct trials. This *choice advantage* indicated that observer’s own actions were sensitive to the attention control state of the actors. The author’s proposed that the greater predictability of the actor’s kinematics in the choice trials allowed observers to respond more quickly.

This hypothesis was put to the test in subsequent experiments in Pesquita et al. (2016). In one condition, when the video recordings were cut to different lengths, observers were able to predict the actor’s reach more accurately on choice trials even before the actor’s had had begun to move. In other conditions, the authors showed that the signal for these predictable movements was available when observers viewed only the actor’s head or only the actor’s torso and limbs. When Pesquita et al. (2016) measured the observers’ social aptitude using the Autism-spectrum Quotient (Baron-Cohen et al., 2001), they found that higher levels of social aptitude were correlated with greater sensitivity to the differences between choice and direct trials.

Chouinard et al. (2024) recently followed up on this work by studying observers over a broader range of the autism spectrum, looking more closely at the kinematics of the observer’s reaches, and by examining in greater detail the differences in the signal strength for the choice advantage among the actors from phase one. This finer-grained analysis revealed choice-advantage in observer responses that were stronger to some actors than others, with autistic observers showing a reliable choice-advantage only in response to one of the actors. Moreover, when the observer kinematics were explored, autistic observers showed sensitivity to this signal only fairly late in the temporal course of their own actions, whereas non-autistic observers showed sensitivity much earlier. These findings highlight the importance of investigating individual differences in action production and how these differences interact with individual differences in observer sensitivity to social signals.

Benedek et al. (2018) studied the conceptually related question of how the signals given by the eyes differ when someone is directing attention internally (e.g., answering from memory) versus when they are directing attention externally (e.g., reading). In phase one, actors were asked to solve anagrams that either remained on view (external attention) or disappeared after 1 s (internal attention), while their faces were video recorded. Previous research by this group had established reliable differences in gaze behavior for these two tasks (Walcher et al., 2017), with internal attention correlated with a greater number of fixations, shorter fixations, and saccades over a larger region of space. Independent observers in phase two viewed randomly ordered videos, still photos, and photos masking the eyes, while trying to discriminate between the two conditions. Observer accuracy was well above chance in the video condition, lower but still above chance in the photo condition, and at chance in the masked eye condition. The authors interpreted this as evidence that observers can access a reliable signal, in the eyes of someone else, of their current attentional direction.

The limitations of the studies in this section are again evident when comparing them to others in Table 1. First, the studies are limited by the relatively small number of actors that have been studied so far. Also, although correlational evidence linking social aptitude to performance is given in the individual differences associated with autism (Pesquita et al., 2016), and in the comparison of autistic and non-autistic observers (Chouinard et al., 2024), a causal link to social factors has not been established. Benedek et al. (2018) did not examine individual differences in either actors or observers. These studies are therefore ripe for experimental manipulation of social influences on performance. A promising avenue for future research is seen in the preliminary data reported from a small phase one sample in Chouinard et al. (2024), showing that the social aptitude of the observer interacts with the individual differences in various actor’s performances.

### 2.3 Attention to other’s intentions

Manera et al. (2011) used an action-observation design to study whether observers could distinguish different social intentions in a reach-to-grasp movement of the hand. They filmed the hands of actors reaching for a block in a tower-building task, in either a cooperative or competitive context. The videos were converted to point light displays in some conditions, to leave only the kinematic motion signals visible to observers. Observers were then shown these video clips in a random order and asked to discriminate whether each one was from a cooperative or competitive trial. Several other non-social conditions were included to control for the speed of the reaches. The results showed that not only were observers able to discriminate between the actor’s intentions, but they could do so in the point light displays, where only kinematic information was available.

Vinton et al. (2024) pursued a related theme by seeking a data-driven answer to the question: Are there fundamental dimensions (latent constructs) that observers used when evaluating the actions of others? These authors used motion-capture technology to record actors performing a large number of everyday tasks (e.g., combing one’s hair, petting a dog, catching a ball). They used these data to animate an avatar that performed these actions devoid of all the idiosyncratic information that normally accompanies someone in action. The observer ratings of these actions were summarized well by two largely-independent factors: friendliness-unfriendliness, and weak-strong. Interestingly, these dimensions resemble the major dimensions used in the evaluation of other’s emotional states (valence, arousal) and also in the evaluation of other’s personalities (affiliative, dominant). The authors interpreted these findings as supporting the view that social perception is guided by the same broad evolutionarily themes in a wide range of domains.

Another hidden construct that guides our attention to the action of others is how prototypical or canonical it appears to be. Brady et al. (2024) used a large set of video clips of everyday actions taken from stock photography sites, which the experimenters categorized into the broad categories of transitive-intransitive (does the action involve an inanimate object?), social-nonsocial (does the action involve another person?), and communicative–non-communicative (is the action intended to communicate to others?). In one set of studies observers were asked to rate how well various still frames in the videos represented the overall movement. A second set of studies showed that in comparison to other frames from the sequences, these ‘canonical moments’ were named more rapidly and accurately, as well as being remembered more accurately.

Table 1 again helps to highlight the limitations of the studies in this section. This line of research seems ripe for studying individual differences in phase one actors as well as in phase two observers. Given the preliminary data in Chouinard et al. (2024), it seems likely that some actors convey their intentions through kinematics more effectively than others, and that some observers are more adept at reading those intentions from the isolated kinematics of hand movements than others. Surely, individuals must also differ in the extent to which they weight the dimensions of friendliness-unfriendliness in assessing other’s actions and in the extent to which they take social dominance (i.e., the weak-strong dimension) into account when doing so.

### 2.4 The perception of emotions while attention is directed to the self or toward others

The aim of Liu et al. (2024) was to study how the social setting influences observers’ selective attention. These authors compared observer’s ability to discriminate facial expressions of emotion when they were self-monitoring and other-monitoring. Self-monitoring refers to individuals observing and regulating their own behavior in an effort to manage their social impressions (Snyder, 1974). While self-monitoring often occurs naturally during social interactions, its influence on social perception is rarely studied.

In phase one, actors were recruited to view emotionally evocative pictures while their faces were video recorded. These pictures, selected from the International Affective Picture database (Lang et al., 2008), varied in both emotional arousal (high vs. low intensity) and valence (positive vs. negative). The actors indicated on each trial whether the images were negative or positive in valence. For half of the recordings, actors were unaware that their emotional expressions were recorded and so any facial expressions they made were spontaneous. For the other half, the actors were instructed to intentionally express the emotion they believed was most appropriate for each picture. These procedures produced a set of video clips that contained both spontaneous and intentional facial expressions, with some in each set resulting in either weak or strong intensity facial expressions of valence. Pilot tests of observers viewing these clips showed that there was overall a highly reliable signal of emotional valence, but that there was also considerable actor variation in the strength of the facial expressions (Enns and Brennan, 2009). Liu et al. (2024) selected the clips of three actors from this set that produced reliably strong facial expressions in the intentional condition, regardless of the strength of these actors’ expressions in the Spontaneous condition.

In phase two of the study, randomly selected video clips of the actors’ facial expressions were presented to an independent set of observers, who were blind to the pictures the actors had seen and were naïve to the conditions under which the facial expressions had been recorded. The observers’ task was to indicate whether the actor was likely viewing a positive or negative picture on a 7-point scale. Some observers were randomly assigned to a self-monitoring condition, where they were told their performance was being evaluated by the actor whose video clips they were viewing. This evaluation would determine whether they would be chosen for a subsequent social game. Other observers were assigned to the other-monitoring condition, where they believed they were evaluating the actor for the same game. Pre-programmed online conversations and activation of the observers’ webcam were included to help reinforce the two monitoring conditions. After the facial expression classification task, the observers reported their subjective stress levels and the believability of the social interaction.

The results showed that the observers were generally sensitive to the affective signals in the facial expressions, with their sensitivity influenced by both the intensity level of the expressions (greater accuracy for high vs. low) and by the type of the facial expressions (greater accuracy spontaneous vs. intentional). But the main finding of the study was that observers were less sensitive to the valence of the expressions when they were self-monitoring, compared to when they were other-monitoring. It was even significantly different when actors’ expressions were made spontaneously. Moreover, this self-monitoring reduction in emotional sensitivity was independent of observers’ self-reported stress. A second experiment in Liu et al. (2024) explored the influence of the believability of the social scenario to induce self-monitoring. These results showed that the self-monitoring reduction in emotional sensitivity was strongest for observers who gave the highest ratings of believability.

This was the only study in this series to include a systematic manipulation of social influence on observers, showing an important link between attention directed to self and the ability to read emotional expressions in others. This manipulation therefore holds promise for studying other social-cognitive abilities. But it also had limitations, in that only a small number of actors were used to create stimulus materials for a large number of observers. This leaves much yet to study, regarding individual differences in the expression of emotions in response to evocative events.

## 3 Actors performing tasks in collaboration, independent observers evaluate their performances

This section reviews three studies that used the action-observation method to examine the production and perception of social collaborations between pairs of people. Each of these studies attempts to mimic, as best one can in the laboratory, the kinds of social interactions people engage in every day. They thus include ‘interactive reciprocity’ (Ristic and Capozzi, 2022) and a ‘second-person’ perspective (Schilbach et al., 2013). By recording these sessions, we are then able to study the perception of these social exchanges by third-party observers. A first study examined the production of New Orleans jazz music by two musical actors in a duet, before studying the auditory perception of these duets by observers. A second study measured the benefits and costs of two actors performing a collaborative visual search task. A third study examined the movement synchrony when pairs of children moved a table through a short maze.

### 3.1 The production and perception of jazz duets

Pesquita et al. (2014) studied the production and perception of social interactions using the microcosm of improvised jazz duets. To create their stimulus materials in phase one of the study, they invited and paid first-rate professional jazz musicians to come to the lab to make playful and engaging recordings of songs they play professionally on a regular basis. These included the New Orleans jazz standards of “Take the A Train,” “Beautiful Love,” and “Canal Street Blues.” The musicians played in time to a click-track, allowing them to maintain uniformity in the tempo of a song. They played each song several times, but each time in a somewhat different way, as occurs when they play professionally and respond to each other in an improvisational way.

Each musician was recorded in a separate soundproof booth, allowing them to hear one another through a live audio feed, but also allowing the researchers to make separate recordings of each instrument. These recordings were then used to construct the three different types of duets that varied in their social interactivity. The condition of greatest interest were the live duets, which allowed for two-way perception (auditory) and action (motor) interactions to occur between the performers. These live duets were compared with dubbed duets, where one musician played along with a prerecorded track of the other musician from a previous recording, without knowledge that the other musician was not live at the moment. Both of these types of duets were then also compared with mix duets, which involved separate live recordings of each musician that had been combined in the studio, by taking a portion of the same song, played by each instrument on a separate occasion. When these duets were played back to the performing musicians, who were asked to rate the quality of the duets, the live performances were rated much more highly than the studio-dubbed duets. This showed that the performing musicians were sensitive to the two-way interactions that were possible in the live recordings, when compared to the one-way interaction of studio-dub tracks. However, somewhat surprisingly, the performers rated the mixed duets even more highly, despite the fact that they had never heard these tracks (recall that they had been created by the study’s authors in the studio). This finding suggests that the performers were not basing their ratings on their personal memory of the recording sessions. It also suggests that the performers were entertained by the novel possibilities inherent in these random track combinations.

In phase two of the study, participants blind to the conditions under which these recordings had been made, and naïve to the genre of New Orleans jazz music, were invited to listen to short samples of the three types of duets in a random order. In one experiment, participants made explicit judgments to indicate the likelihood that each track was a live versus studio recording. The results showed that a majority of listeners were able to discriminate the live tracks from the studio tracks. The authors interpreted this finding as supporting the hypothesis that social intelligence helps listeners to understand the collaborative nature of jazz, even when they are unfamiliar with the genre.

In a second experiment in the same study, a different group of participants used rating scales to judge the recordings on four dimensions of musicality: emotionality, engagement, creativity, and synergy. Following the listening session, participants provided background information about their general social aptitude (Autism Spectrum Quotient) and their personal history of musical training (Musical Expertise Questionnaire). The results showed that among listeners with the least musical training, this sensitivity was correlated with their social aptitude scores. The authors using these findings to argue that the human ability to assess the quality of a social interaction (Blakemore and Decety, 2001) is present even when the interaction is auditory, nonverbal, and in a medium in which the listeners themselves are not skilled. They claimed the results pointed to an important link between social aptitude and the ability to perceive the quality of a musical interaction (Phillips-Silver and Keller, 2012).

In a third experiment (not included in the original article) participants were asked to simply tap their fingers in response to the beat of the music in each track. Once again, the three types of duets were presented randomly. The results here showed that the variability in their tapping was directly linked to the possibility of social interaction in the recorded music. Tapping was most regular (least variable) for the live duets on average, somewhat less regular during the studio-mix tracks, and least regular for the studio-mix tracks. The author’s interpretation of these motor action measure was that even the body can tell when the intangible synergy of social collaboration adds value to the music we are listening to.

This study is unique in that the actors were professionals who engage in social interactions for their livelihood. Although many individuals are experts in the everyday social exchanges of daily life, only a few have trained for years to engage in social exchanges that listeners appreciate as art. This honing of a craft over a career may have contributed to the ease with which naïve listeners were able to hear the difference between music performed in a live two-way interaction versus music that was only one-way or an artificial construction. The small sample size of actors in this study, however, prevented analysis of individual differences in the ability to create music that was more engaging when played live than when only one or none of the actors had the ability to respond to the other musician.

### 3.2 Collaborative visual search

Brennan and Enns (2015a) conducted a study of collaborative cognition, which refers to solving a problem or making a decision together with others. Collaborative cognition occurs regularly in everyday activities such as looking at a website together with another person in planning travel or in navigating together in car to an unfamiliar location. Their study was motivated by the observation that although collaborative cognition often leads to better problem-solving and decision-making outcomes than individual cognition, the specific conditions that maximize these benefits are not well understood. The focus of Brennan and Enns (2015a) was to develop measures that would help distinguish between the collaborative benefits that arise from synergistic interaction between partners versus the benefits that result from simply pooling individual efforts.

In phase one of the study, pairs of participants completed a visual enumeration task. Sitting side by side, the participants who viewed a busy display of common objects were given the task of deciding how many of four potential target objects were in each display (baseball, apple, coffee can, toy penguin). These objects appeared randomly in one of many locations on each trial and the number of target objects displayed varied randomly between 0, 1, and 2. Participants took turns keying in their answers. The main dependent variable was the speed with which the correct answer was indicated, since accuracy in this task was very high (more than 90% correct).

To help compare the results of each team’s performance with their individual abilities, each participant was tested in an individual condition and as a member of a two-person team. The results showed that team performance on average exceeded the efficiency of two individuals working independently, indicating that interpersonal interaction underlies the collaborative gains in this task. However, there was also significant variation in this team efficiency, with most teams showing the benefit over combined individual performances but a few teams not showing this benefit, and a few even showing performance costs of working together. A subsequent study examined the correlations between these performance differences between teams and various measures of brain synchronization of team members, both synchronization between regions within individual brains, and phase synchronization between the same brain regions in team partners (Szymanski et al., 2017). The results suggested that phase synchronization between brains constitutes a neural correlate of social facilitation, helping to explain why some teams performed better than others.

In phase two of Brennan and Enns (2015a), this variation in team performance was examined behaviorally in two different ways. The variables in this phase examined both the strength of the social bond experienced by the members in each team and by the similarity in verbal behavior that team members used to communicate with one another. The strength of the social affiliation experienced by team members was first assessed by having participants complete the Intimate Friendship Scale (Sharabany, 1974), which consists of questions such as “I can be sure that my friend will help me whenever I ask for it.” Responses were made using a 5-point Likert scale, with each team score consisting of the sum of the two individual scores. An independent measure of social affiliation was achieved by having observers, blind to the purpose of the experiment, viewing video clips of the two participants on each trial, taken from the computer’s camera at the top of the visual display. This measure was not reported in Brennan and Enns (2015a), because at the time it was considered redundant with the participants’ own ratings of their affiliation strength (it correlated strongly, *r* > 0.80). For the purposes of this review, however, it helps to emphasize the point that naïve observers are sensitive to the signals of affiliation visible in brief video clips of participants working together to make a decision.

The verbal behavior exhibited by team members was assessed in phase two by an observer blind to the purpose of the study. This individual viewed the video clips of each trial in phase one of the study and made a written transcript of all verbal communication during the team task. This included both the content of each utterance and the total number of distinct utterances made by each team member. The total number of different utterances made by participants were tallied for each team (content) and this sum was divided by the total number of team utterances. This generated an index of communication similarity, in which smaller scores indicated greater similarity.

A regression analyses examined the ability to predict the actors’ phase one decision time based on these two measures from the phase two observations. The results showed that teams who reported stronger affiliation (*p*r = 0.346) and communicated more similarly (*p*r = 0.502) were also those who showed greater collaborative benefits. The partial correlation between these two predictors were near zero (*p*r = 0.048), and so taken together, these two predictors of collaborative efficiency accounted for 39% of the total variance in team performance (*p* < 0.001). The authors concluded that the benefits of collaborative cognition are influenced by at least two independent factors: the strength of social affiliation between members and by the similarity of their verbal communication.

In a follow-up to this initial study, Brennan and Enns (2015b) examined the possible contributions of affiliation strength and communication style to collaborative cognition in greater detail. They employed the same visual enumeration task as in the original study, but this time they controlled these two variables more deliberately. To control affiliation strength, they recruited pairs of friends to the study, but then assigned half of the participants to complete the enumeration task with the friend they had brought and the other half of the participants to complete the task with someone else’s friend (i.e., a stranger). To control for the non-verbal aspects of communication, they assigned half of the teams to complete the task as in the original study (full visibility) and the other half of the teams to complete the task with a visual barrier that blocked vision of the partner’s face and torso.

The results from phase one of the study measured each team’s collaborative efficiency in the same way as the original study, but this time the efficiency score for each team was compared in a 2 × 2 design, involving teams of friends versus strangers and of teams with full visibility of one another and those whose vision of the partner was blocked by a partition. The outcome was a strong interaction between the variables of friendship and partner visibility. Friends generally collaborated more efficiently than non-friends when visible to one another, just as in the original study. However, when a partition prevented pair members from seeing one another, their collaborative efficiency of friends was reduced to the same lower level as strangers. The authors interpreted this as indicating the importance of subtle visual and non-verbal signals that friends have learned to use to communicate efficiently. A secondary finding of this study was that pairs of female friends tended to perform the enumeration task more efficiently than pairs of male friends, though this effect was independent of the overall interaction between friendship and visibility.

In phase two of the study, Brennan and Enns (2015b) examined the quantity of verbal communications made by these four types of teams, using similar procedures as in the original study. Here their novel finding was that when partners were visible to one another, the overall quantity of utterances was similar between friends and strangers. However, when partners were separated by a visual barrier, the number of utterances made between friends rose abruptly. It was as though the absence of a visual communication channel led to the increase in the perceived need for friends to communicate verbally. Coincidentally, this was accompanied by their reduced task efficiency in phase one of the study. The authors interpreted this finding as illustrating the natural trade-off that exists between the costs in decision time of increased communication, versus the benefits to decision time of having rapid non-verbal channels of communication. In this case, those benefits seem to have arisen from the familiarity and/or predictability in communication that occurs in a longer-term friendship.

These studies of collaborative visual search are clearly limited, when compared to the other studies, in their small sample sizes for the observers (Table 1). The stronger emphasis in these studies was on the characteristics of the actors and the settings that allowed pairs of participants to outperform the prediction generated by considering each of their performances in isolation. Clearly, much more can be done in the future to understand the observer characteristics and observer settings that allow for more accurate perception of the social synergy that can arise when people work together in teams.

### 3.3 Collaborative joint action

Trevisan et al. (2021) conducted a study of joint action, in which they invited pairs of children with autism spectrum disorder (ASD) to move small tables through a simple maze in a classroom. In the first phase, each child lifted one end of a table that was too large to carry alone and maneuvered it with the other child through an S-shaped path between other tables. The researchers hypothesized that children with social communication difficulties would show less joint-action coordination than neurotypical children.

They measured action coordination using an iPhone strapped to the underside of the table, programmed to record the duration of the carrying event and the amount of “wiggle” in three dimensions. The study compared these measures in pairs of children with ASD to pairs of neurotypical children matched in non-verbal intelligence scores. Additionally, both sets of children carried the same table through the maze with an adult experimenter to examine their joint action capabilities with an adult providing social “scaffolding.” Each child also carried a smaller table through the maze alone to provide a baseline measure of individual action coordination and control for any gross motor perturbations.

Results from phase one showed that tables carried by children with ASD took longer to move and wiggled more than those carried by neurotypical children, even after controlling for individual table-carrying abilities. However, these differences disappeared when children carried the table with an adult, highlighting the benefits of one member of an action pair compensating for the other’s actions.

In phase two, an independent adult observer watched videos of the table-carrying episodes. The observer, blind to the study’s purpose and the children’s social communication abilities, viewed videos recorded from multiple synchronized camera angles using specialized software. The observer categorized each video into one of three categories: (a) In-sync, indicating that participants’ legs moved in unison, (b) Out-of-sync, indicating no clear coordination between participants’ steps, and (c) Legs-out-of-sight, indicating that the video footage did not capture an interpretable view of the participants’ legs. A second adult observer coded 10% of the videos to estimate reliability, yielding an 82.7% agreement. The researchers calculated a stepping synchrony score by creating a ratio of in-step episodes to the total remaining episodes, adjusted for the duration of each episode to control for group differences in episode length.

Results from phase two showed that the proportion of time spent in synchronous stepping was high for both groups when carrying the table with an adult (mean = 0.83). This proportion fell to 0.65 for pairs of neurotypical children and to 0.37 for pairs of children with ASD. Trevisan et al. (2021) interpreted these findings to show that the coordination of joint action is a highly adaptive social process involving synchronization of both mind and body. This synchronization is atypical in children with ASD, even when accounting for differences in individual motor abilities. However, these difficulties can be overcome when a joint action partner, like an adult, compensates for these challenges.

This study, like the studies of collaborative visual search, were limited by the very small sample sizes of observers. Future studies will therefore be again needed to investigate the observer characteristics and observer settings that allow for accurate perception of the social synergy that can arise when people work together in teams.

## 4 Future directions

This review has summarized the action-observation methodology for studying the interaction that occurs between the *production* of actions in social settings and the *perception* of these actions by others. We have provided readers with example studies from our lab and others that illustrate the implementation of this methodology. It is clear from the comparisons among studies in Table 1, that none of these studies have mined the full range of possibilities in this methodology. Some of the studies would clearly benefit from closer examination of the factors influencing observer perception; other studies would benefit from systematic study of the influences and individual differences that affect actor behavior. However, the two lines of research we have summarized — one focusing on the perception of individual actors performing a task and the other investigating the perception of collaborating pairs of individuals — illustrate the broad range of application that are possible with this methodology.

The studies examining the perception of solitary actors performing tasks gave important insights on how people model the attention and the intention of others. To recap briefly, Brennan et al. (2011) showed that observers who were naïve to what actors were seeing were nonetheless sensitive to these actors’ attentional effort as they performed their searches. The observers’ models of attentional effort appeared to be informed by the signals conveyed in the actors’ eye movements, head movements and facial expressions of emotion. The studies by Pesquita et al. (2016) and Benedek et al. (2018) demonstrated that observers’ mental model of the actors’ attention included information about the actors’ state of attention control and whether it was focused externally or internally. Both studies showed that observers’ responses to the actors’ actions were influenced by whether actors were guided by external signals or by internally made choices. Moreover, observer sensitivity to this distinction in Pesquita et al. (2016) was correlated with measures of social aptitude, as indexed by the autism quotient rating scale in the general population. The autism diagnosis status of the observers was explored in greater detail by Chouinard et al. (2024). Manera et al. (2011) showed that the observer’s model of others’ action contains information about their social intentions. Vinton et al. (2024) and Brady et al. (2024) showed further that the formation of these models is guided by latent constructs, such as friendliness-unfriendliness, weak-strong, and prototypical movements. The focus of Liu et al. (2024) was on how attention to the self in a social setting, when compared to attention to others, reduces observers’ sensitivity to the affective signals of actors’ facial expressions.

Taken together, these illustrations of the action-observation method used to study attention in person perception demonstrate that observers can often model others’ attention and intention with considerable accuracy. These internal models include information such as the cognitive effort, the actor’s state of attention control, whether the actor’s direction of attention is internal or external, and whether the action is in a cooperative or competitive social setting. Liu et al. (2024) took an additional step in showing that these models are influenced by the observer’s own social context.

### 4.1 A possible future study of person perception

One potential future direction in this line of research could use the action-observation method to examine the broader context in which actors and observers are embedded. For example, a recent comprehensive review of social factors influencing the joint orienting of attention reported that whether one shifts one’s gaze in the direction of another person’s gaze depends not only on characteristics of the participant (e.g., gender and personality), but also on situational factors such as the familiarity of the other person, their affiliation to the participant, and the perceived naturalness of the social exchange (Dalmaso et al., 2020).

One example of possible research on these issues might begin with the observation that people frequently alternate between locking gaze with their conversation partner and then looking away. These *looks away*, sometimes referred to as gaze aversion, are thought to signal more than one kind of attention shift. On some occasions, a conversation partner looks away in order to gather information from the surrounding environment or to provide a reference in the environment for joint attention (Bayliss et al., 2013; Ricciardelli et al., 2009). This type of looking away is therefore a potential signal of externally-directed attention in one’s conversation partner. On other occasions, a conversation partner looks away in order to reduce their cognitive load while thinking over the answer to a question posed by their partner (Doherty-Sneddon and Phelps, 2005). The hypothesis here is that reading social information from a face and answering a question are in conflict because they each require the same mental resources in working memory. This type of looking away is therefore a potential signal of internally-directed attention (Benedek et al., 2018), which can serve as a social signal to hold one’s place in a conversation while thinking (Morency et al., 2006). If these two types of gaze aversion convey different intentions of the interlocutor, it would clearly be advantageous for people to differentiate between them in everyday conversation, in order to facilitate a fluent and effective social exchange during conversation. To date, some of the factors influencing the *production* of gaze aversion have been studied (Maran et al., 2021; Bianchi et al., 2020), along with some physical differences between these two types of gaze aversion. However, only a handful of studies have investigated the perception of gaze aversion in others (Jording et al., 2019; Morency et al., 2006; Servais et al., 2022), and none have so far studied their production and perception in the same study.

The action-observation methodology lends itself well to this question. In phase one, one might recruit actors in order to record instances of external versus internal directed gaze aversions during the course of a natural conversation. The spatial extend of the actors’ eye movements (e.g., number of fixations, dwell time, scan paths) and head movements could be measured using mobile eye and head trackers. Differences between these two types of gaze aversion could be documented in order to understand the signals that a conversation partner might use to do the same. In phase two, observers could be presented with video recordings of the actors’ behavior during the conversation, after removing contextual information to indicate whether the prompt for the look away was externally or internally motivated. This could be done by audio-muting the video recordings and editing them for consistent durations across the two conditions. The data from the observers in phase two would then allow an assessment of observer’ accuracy in discriminating the two types of gaze aversions. In addition, this accuracy measure could be used in conjunction with the physical differences documented in phase one, to determine how the signals are informed by the various eye and head movement features. Individual differences in both the actors’ expression of these two types of gaze aversion and in the observers’ sensitivity to these differences could also be examined.

The second line of studies reviewed in this paper investigated the production and perception of collaborative social interactions. By designing tasks that closely mimic everyday social exchanges, these studies explored the factors influencing the performance of collaborative tasks in a more naturalistic environment. Moreover, these studies also show that the efficiency of a social interaction relies heavily on each individual’s access to bodily cues from their partners. Specifically, Pesquita et al. (2014) showed that jazz musicians use auditory and visual cues to continuously predict and adapt to each other’s action. When these interactive exchanges are interrupted (e.g., when a musician is playing with a pre-recorded track), naïve observers assess the interaction to be lower in musical quality. Similarly, in Brennan and Enns’s (2015a, 2015b) study, the efficiency of a joint visual search task was influenced by access to non-verbal cues of the partners and by the social affiliation of the collaborating partners. Trevisan et al. (2021) demonstrated that individuals with social communication difficulties were less able to synchronize their action with others, resulting in less efficient performance during joint action.

### 4.2 A possible future study of collaborative action

One way to continue the study of collaborative action is to examine the production and perception of a partner’s effort in a joint task. For example, Chennells and Michael (2018) reported that individuals performed better in a tedious key pressing task when they thought their task partner was exerting high levels of mental effort. However, joint action tasks often require partners to contribute some combination of both mental effort and physical exertion for the task to be accomplished. To date, there have been very few studies focused on how the perception of a partner’s physical exertion influences one’s own physical performance. This despite the fact that exercising together is something that many people do and enjoy on a daily basis. The research done to date has focused primarily on the accuracy of exertion perception in others by passive observers (e.g., coaches of athletes and onlookers; Paul et al., 2021). No study to date has investigated the perception of exertion in others when both the actor and observer are exercising at the same time, making them both ‘second-person interactors’ (Schilbach et al., 2013).

This question could be investigated using the action-observation method. In phase one, pairs of participants that are similar in their physical fitness level, gender, and age could be instructed to complete short bouts of cycling exercise in the same room, in front of a mirror, so that they have full visibility of one another. The exercise bouts might include a combination of intensity levels for each participant, so that in some bouts they are exercising at similar levels of intensity and in other bouts the intensities contrast with one another. These actor participants would be video recorded during the exercise sessions so that their joint performances could be viewed and assessed by observers in phase two that are blind to the factors influencing their different levels of intensity.

After each bout of joint exercise in phase one, participants could be asked to rate their own level of physical exertion and that of the exercise partner. These ratings could then be analyzed and compared to participants’ objective levels of exercise intensity (e.g., cycling wattage). Additional measures of each participants’ experience could also be gathered in phase one (e.g., heart rate, self-reports of affect, motivation, and exercise satisfaction) to further explore their relationship with self-and other-ratings of exertion.

In phase two, the video clips recorded in phase one could be presented to a set of observers, blind to the measures taken in phase one, who might be asked to make assessments of both individual and joint performances. This would help to identity the sources of information observers use when making judgments of exertion in others as passive onlookers. The bodily sources of these signals could also be studied by masking regions of the video recordings (e.g., showing only the head or the torso of the actors).

In conclusion, we offer this review as an illustration of how the action-observation methodology has been used in the past, the potential of this methodology to go well beyond how it has been used so far, and the potential of this methodology to open up new avenues of study in the social-cognitive realm. Minimally, we hope that outlining this method and its potential uses will inspire future research in an effort to deepen our understanding of social perception.
